# Bilingualism and cognitive reserve: Preserving cortical thickness in aging individuals

**DOI:** 10.1002/alz70857_103108

**Published:** 2025-12-24

**Authors:** Tanya Dash, Yves Joanette, Ana‐Inès Ansaldo

**Affiliations:** ^1^ University of Alberta, Edmonton, AB, Canada; ^2^ Canada Institutes of Health Research, Montreal, QC, Canada; ^3^ University of Montreal, Montreal, QC, Canada; ^4^ IUGM research center, Montréal, QC, Canada

## Abstract

**Background:**

This study investigates the relationship between bilingualism, cognitive reserve, and cortical thickness in aging bilingual individuals. It explores the potential role of bilingualism in preserving brain structure against age‐related decline. Cognitive reserve was evaluated using established measures of bilingualism (Dash et al., 2022) and the cognitive reserve index (CRIq; Nucci et al., 2012), accounting for gender effects to minimize bias.

**Method:**

A total of 79 bilingual individuals, aged 30 to 79 years, underwent neuroimaging analysis to assess cortical thickness using the FreeSurfer atlas. Partial least squares regression (PLSR) was employed to examine the relationship between age, bilingualism, and cognitive reserve indicators (such as CRIq) and cortical thickness across several brain regions.

**Results:**

The analysis revealed three latent variables (LVs) that elucidate key brain‐behavior relationships:

The first latent variable, primarily driven by age, was associated with cortical thinning in regions typically impacted by aging, including the bilateral middle temporal cortex, hippocampus, lateral orbitofrontal cortex, and inferior temporal cortex.

The second latent variable, incorporating both age and CRIq as predictors, highlighted regions linked to cognitive control and sensory processing, such as the insula, thalamus, and superior frontal cortex, indicating a protective effect of cognitive reserve against age‐related cortical thinning.

The third latent variable, with bilingualism‐related measures as the predictor, revealed preserved cortical thickness in regions associated with language and executive control, including the pars opercularis, orbitofrontal cortex, and inferior parietal cortex, suggesting that bilingualism may provide a neural shield against aging‐related brain changes.

**Conclusion:**

These findings support the hypothesis that bilingualism and cognitive reserve contribute to the preservation of cortical thickness in key regions, particularly those involved in cognitive control, language, and executive functions. This study provides further evidence for the protective role of bilingualism in maintaining brain structure and function in aging, contributing to the broader understanding of cognitive reserve mechanisms.

